# Buoyancy under Control: Underwater Locomotor Performance in a Deep Diving Seabird Suggests Respiratory Strategies for Reducing Foraging Effort

**DOI:** 10.1371/journal.pone.0009839

**Published:** 2010-03-23

**Authors:** Timothée R. Cook, Akiko Kato, Hideji Tanaka, Yan Ropert-Coudert, Charles-André Bost

**Affiliations:** 1 DST/NRF Centre of Excellence, Percy FitzPatrick Institute of African Ornithology, University of Cape Town, Cape Town, South Africa; 2 Centre d'Études Biologiques de Chizé (CEBC), CNRS UPR 1934, Villiers-en-bois, France; 3 National Institute of Polar Research, Tokyo, Japan; 4 Institut Pluridisciplinaire Hubert Curien (IPHC), Département Écologie, Physiologie et Éthologie (DEPE), Université Louis Pasteur - CNRS, Strasbourg, France; 5 COE for Neo-Science of Natural History, Graduate School of Fisheries Sciences, Hokkaido University, Hakodate, Hokkaido, Japan; University of Queensland, Australia

## Abstract

**Background:**

Because they have air stored in many body compartments, diving seabirds are expected to exhibit efficient behavioural strategies for reducing costs related to buoyancy control. We study the underwater locomotor activity of a deep-diving species from the Cormorant family (Kerguelen shag) and report locomotor adjustments to the change of buoyancy with depth.

**Methodology/Principal Findings:**

Using accelerometers, we show that during both the descent and ascent phases of dives, shags modelled their acceleration and stroking activity on the natural variation of buoyancy with depth. For example, during the descent phase, birds increased swim speed with depth. But in parallel, and with a decay constant similar to the one in the equation explaining the decrease of buoyancy with depth, they decreased foot-stroke frequency exponentially, a behaviour that enables birds to reduce oxygen consumption. During ascent, birds also reduced locomotor cost by ascending passively. We considered the depth at which they started gliding as a proxy to their depth of neutral buoyancy. This depth increased with maximum dive depth. As an explanation for this, we propose that shags adjust their buoyancy to depth by varying the amount of respiratory air they dive with.

**Conclusions/Significance:**

Calculations based on known values of stored body oxygen volumes and on deep-diving metabolic rates in avian divers suggest that the variations of volume of respiratory oxygen associated with a respiration mediated buoyancy control only influence aerobic dive duration moderately. Therefore, we propose that an advantage in cormorants - as in other families of diving seabirds - of respiratory air volume adjustment upon diving could be related less to increasing time of submergence, through an increased volume of body oxygen stores, than to reducing the locomotor costs of buoyancy control.

## Introduction

Air-breathing vertebrates have attained underwater feeding grounds, exploiting rich food patches through morphological and physiological adaptations to diving. The efficiency of most divers in exploiting underwater resources depends on their oxygen (O_2_) supplies and their capacity to reduce heat loss to the aquatic environment [1]. However, the capacity for divers to control the force of buoyancy is also a crucial parameter affecting diving efficiency [2], [3].

Hereafter, we define buoyancy as the vertical force acting on a submerged animal and that is the difference between the upward force of buoyancy and the downward force of gravity (this is also called “net buoyancy”, Appendix S1). For a diver, the force of buoyancy depends mainly on the amount of air it carries. Indeed, because of Boyle-Mariotte's gas law, the volume of any body air compartment will decrease exponentially with depth under the effect of increasing hydrostatic pressure. Swimming against a strong positive buoyancy during the downward phase of the dive or rising in the absence of positive buoyancy during the upward phase of a dive can mean spending a considerable amount of O_2_, which cannot be allocated to prey searching, capturing or handling.

Divers should thus exhibit behavioural strategies for reducing the O_2_ consumption of buoyancy control. For years, the examination of these strategies has been challenging because of the difficulties in observing submerged diving animals [4]. Bio-logging now enables monitoring of underwater animal behaviour, and the present issue is to analyse and interpret the vast amounts of information that are recorded with data-loggers. The study of underwater activity using cameras [5]–[7], Hall sensors [8], acoustic data-loggers [9], [10] or data-loggers recording acceleration (accelerometers) [11]–[15], has revealed that several marine mammals, turtles and birds adjust tail-, flipper-, wing- or foot-stroke frequency and amplitude in relation to depth. Such behavioural patterns reflect the way the force of buoyancy on the animal decreases naturally with depth (if the animal does not expel a substantial portion of respiratory air while swimming). Some seals and cetaceans will cease tail beating at some point during the descent, thus gliding the rest of the way to maximum dive depth [5], [11]. Turtles will decrease flipper beat frequency with depth [7]. Penguins and alcids will cease flipper or wing activity at some point during the ascent, using the growing force of buoyancy to ascend passively [13], [14]. Because O_2_ consumption rate is directly related to locomotor activity [16], any strategy that will reduce this activity can be considered as an O_2_ saving strategy.

Seabirds are particularly affected by the problem of managing the force of buoyancy. Indeed, they load large amounts of air upon diving, air which is present in their plumage, in their bones, and in their large respiratory system comprised of air sacs. Cormorants are flying, foot-propelled divers that feed predominantly on prey near the water bottom. Previous studies investigating the underwater locomotor activity of cormorants have focused on shallow diving species, which probably do not dive deeper than their depth of neutral buoyancy [15], [17]–[20]. The depth of neutral buoyancy can be defined as the depth at which the hydrostatic pressure has reduced the volume of body air compartments to the point where the buoyancy and the gravity acting on the body cancel each other out (net buoyancy  = 0). In the present study, the underwater locomotor activity of a deep-diving cormorant, the Kerguelen shag *Phalacrocorax verrucosus*, is explored, using miniaturized accelerometers. These data-loggers, when deployed on an animal, will record the accelerations made over time by the animal's body in one, two or three dimensions. If appropriately calibrated, such accelerations can be used to describe the animal's body movements, therefore its behaviour [21] or its rate of energy expenditure [18]. Because cormorants are foot-propelled divers, an important component of acceleration is the surge acceleration, the one that occurs in the head-to-tail axis [22].

Kerguelen shags are deep-diving long-lived seabirds, which feed close to the sea bottom mainly on benthic fish from the Notothenoid sub-order [23]. Deep benthic diving necessitates careful management of O_2_ stores, so that bottom duration, i.e. foraging duration, will last as long as possible. By deploying accelerometers on free-ranging Kerguelen shags, we aimed at studying the locomotor and any possible respiratory strategies of deep-diving cormorants for reducing the underwater swimming effort of buoyancy control. Our goal was: 1) to determine if birds adjust their locomotor activity to variation in the force of buoyancy with depth, thus saving energy, 2) to investigate whether birds rise passively at some point during the ascent phase, suggesting they might glide to the surface after crossing their depth zone of neutral buoyancy [24], and 3) to explore what parameters influence the depth zone of neutral buoyancy in Kerguelen shags. In particular, because deeper dives last longer, cormorants can be expected to load more air, therefore more O_2_, when they dive to greater depths. Because this question is actually difficult to test on free-ranging birds, it has received to date little attention in the literature (but see: [11], [25], [26]). In the Kerguelen shag, we tested whether the depth zone of neutral buoyancy increased with increasing maximum dive depth, this relationship being a possible proxy for an increase in the volume of respiratory air loaded for deeper diving.

## Methods

### Ethics statement

Animals in this study were cared for in accordance with the guidelines of the ethics committee of the French Polar Institute (Institut Paul Emile Victor – IPEV).

### Study birds and colonies

The study was carried out at Kerguelen Islands, Southern Ocean, during the breeding season, between December 24^th^ 2005 and January 5^th^ 2006. Ten chick-rearing adult Kerguelen shags were captured on their nest with a noose attached to a fishing pole. They were set free after data-loggers were attached to the feathers of the bottom part of the back, using Tesa® tape secured with cyanoacrylate glue (Loctite® 401) (handling time: 10 min max.). During the process, the head was covered with a hood in order to reduce stress due to handling. Animals were recaptured after one day. They were weighed and loggers were removed and data were downloaded to a computer.

The two different study colonies were distant from each other by 30 km: the Sourcils Noirs colony (49° 40′ S, 70° 15′ E) and the Pointe Suzanne colony (49° 25′ S, 70° 25′ E) (Table 1). These colonies are adjacent to two distinct oceanographic environments. Birds from the first colony are larger and forage in deeper waters than birds from the second [23]. Within a colony, males are larger than females and dive deeper than females. As a result of sampling the two sexes in two sites, we expected to obtain dive profiles ranging from shallow to very deep, thus potentially encompassing the shags' depth zone of neutral buoyancy.

**Table 1:** Mean maximum dive depth, mean dive duration and mean bottom duration for the 10 study birds (± S.D.).

Colony	Sex	Body mass (kg)	Number of dives	Dive depth (m)	Dive duration (s)	Bottom duration (s)
PS	f	2.03	34	21.3±3.3	87.6±18.4	64.1±15.6
PS	f	1.98	102	23.2±6.9	105.9±33.3	79.2±26.2
PS	f	2.08	88	37.4±6.1	148.7±26.9	105.1±21.2
SN	f	2.61	37	84.0±24.1	266.8±85.1	169.8±60.9
SN	m	3.16	16	103.3±25	302.9±74.7	174.1±46.9
SN	m	2.97	15	101.5±5.5	304.1±17	187.9±18.2
SN	f	2.77	22	80.4±26.1	242±79.7	143.4±54.1
SN	m	2.82	12	65.2±19.3	182.3±61	103.5±36.1
SN	f	2.60	29	89.9±18.4	274.9±56.3	164.5±35.4
SN	f	2.85	20	108.9±3	289.4±15.2	156.9±15.2

PS  =  Pointe Suzanne.

SN  =  Sourcils Noirs.s

### Data-loggers

Data-loggers were M190-D2GT (Little Leonardo, Tokyo, Japan). These are cylindrically shaped and streamlined (domed heads) in order to reduce hydrodynamic drag [27]. The position of data-loggers on the back of birds was lengthwise relative to body axis, with the domed head facing the direction of the head of the animal. Loggers measured 1.5×5.2 cm and weighed 16 g in air (0.6% of mean body mass, which is below the 3%pecified for flying birds, [28]). Loggers stored data in a 128 Mbit flash memory. Depth was measured every 1 s (1 Hz) by a piezoresistive pressure sensor, with a resolution of ±0.05 m. Tail-to-head acceleration (surge) was recorded every 0.015625 s (64 Hz) by a capacitive accelerometer, with a resolution of 0.0196 m.s^−2^. This sensor measures both dynamic (e.g. propulsion behaviour resulting from body movements) and static (e.g. gravity) accelerations.

### Data analysis

Data were analyzed using a custom-written program in IGOR Pro (WaveMetrics, Version 5.03J). Dives were considered to occur when they were ≥1 m deep. Each dive was divided into three different phases: the descent, the bottom and the ascent. Vertical transit rates were calculated as the vertical speed of descent or ascent. The bottom phase (time dedicated to prey search and capture in a benthic forager) was defined as starting and ending when the vertical transit rate of the bird became <0.25 m.s^−1^ (end of descent) and >0.25 m.s^−1^ (beginning of ascent) [15].

The acceleration studied and discussed here is the head-to-tail acceleration (surge) due to propulsive body movements (feet kicking), from which gravity was subtracted using a two band low-pass filter (0.5/1.0 Hz, [15]). All analyses were conducted following correction for the attachment angle of the logger: body angle (arcsine [gravity acceleration]) was considered to be 0° when the bird rested on the water surface. Swim speed (vertical transit rate/sine [body angle]) was calculated assuming the bird's dive angle corresponded to the longitudinal axis of the bird (head-to-tail). Because swim speed is calculated using vertical transit rate and body angle, speed data were excluded when vertical transit rate was too small (<1 m.s^−1^). This occurs near the end of the descent phase, as the body angle of shags becomes shallower the closer they get to the bottom.

During the paddling cycle, the body accelerates forward during the foot-stroke (cormorants stroke both feet simultaneously) and decelerates during the glide and recovery of the foot (burst and glide gait, [22]). Because of this, a positive acceleration peak is recorded during the stroke [17], [29]. The surge acceleration referred to hereafter is the value of the acceleration at this peak. It therefore represents the acceleration produced by each foot stroke (stroke amplitude). Stroke frequency is defined as the number of strokes per second.

### Statistics

Several parameters can affect buoyancy. Food load will modify buoyancy through modification of the bird's density. A diving seabird's density can be expected to increase between the first and the last dive of a foraging trip with the ingestion of prey. Buoyancy should also depend on body size. Body surface increases less rapidly than body volume when body mass is increased. Consequently, smaller individuals can be expected to load proportionately more air in their plumage relative to their body mass compared to larger ones, when they dive. At Kerguelen, there are two correlates of body size: colony origin and sex (see above). Plumage soaking can possibly be considered as affecting buoyancy. Cormorants are known to have partially wettable plumage [30], [31] and it has been shown in great cormorants *Phalacrocorax carbo* that the partial soaking of the plumage increases with time spent by the bird in the water [32].

We tested the possible effect of these different parameters using a general linear regression model with mixed-effects fitted by restricted maximum likelihood [33]. For every individual, a number was allocated to each dive according to its timing of occurrence inside the foraging trip. The first dive was allocated the value of 1, the second dive the value of 2 and so on. We called this parameter the dive order. Dive order was considered to influence food load and potentially plumage soaking. We therefore tested the effect of dive order and body size (using body mass) on the depth of last foot stroke during the ascent phase of dives. On one hand, maximum dive depth depends on body size. On the other, depth of last foot-stroke may depend on maximum dive depth, assuming birds load more air when diving deeper, therefore longer. Because of this, maximum dive depth was always set as a covariate. Individual was always set as a random factor. Because maximum dive depth of shags must be deeper than their depth of neutral buoyancy for tests to be conclusive, tests were only performed on dives of birds from the Sourcils Noirs colony (excluding 8 dives <10 m deep). The significance of the study parameters was obtained after performing an ANOVA on the models. The analysis was performed using R software [34].

## Results

**Figure 1:**
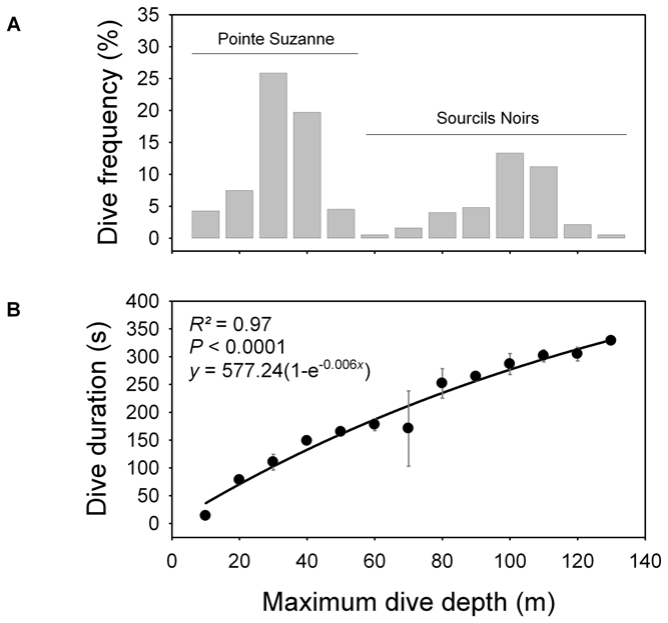
(A) Frequency distribution of maximum dive depths and (B) mean dive duration in relation to dive depth. Shallow dives belong to birds from the Pointe Suzanne colony and deep dives to birds from the Sourcils Noirs colony. Inside each depth class, each dive duration value was calculated as the mean of individual means (± S.D.).

For each bird, accelerometers recorded the complete foraging activity for one day. Foraging consisted of flying at sea to foraging grounds and alternating underwater diving periods with surface recovery periods and then returning to the colony. All birds made a single foraging trip per day, except one bird from Pointe Suzanne which made two. Overall, a total of 375 dives were recorded. Dive depth, dive duration and bottom duration ranged between 1.4–125.5 m, 2–346 s and 0–219 s, respectively (Table 1). Maximum dive depths were distributed according to two main modes: shallow dives (birds from the Pointe Suzanne colony) and deep dives (birds from the Sourcils Noirs colony) (Fig. 1). Overall, dive duration increased with dive depth (Fig. 1).

**Figure 2:**
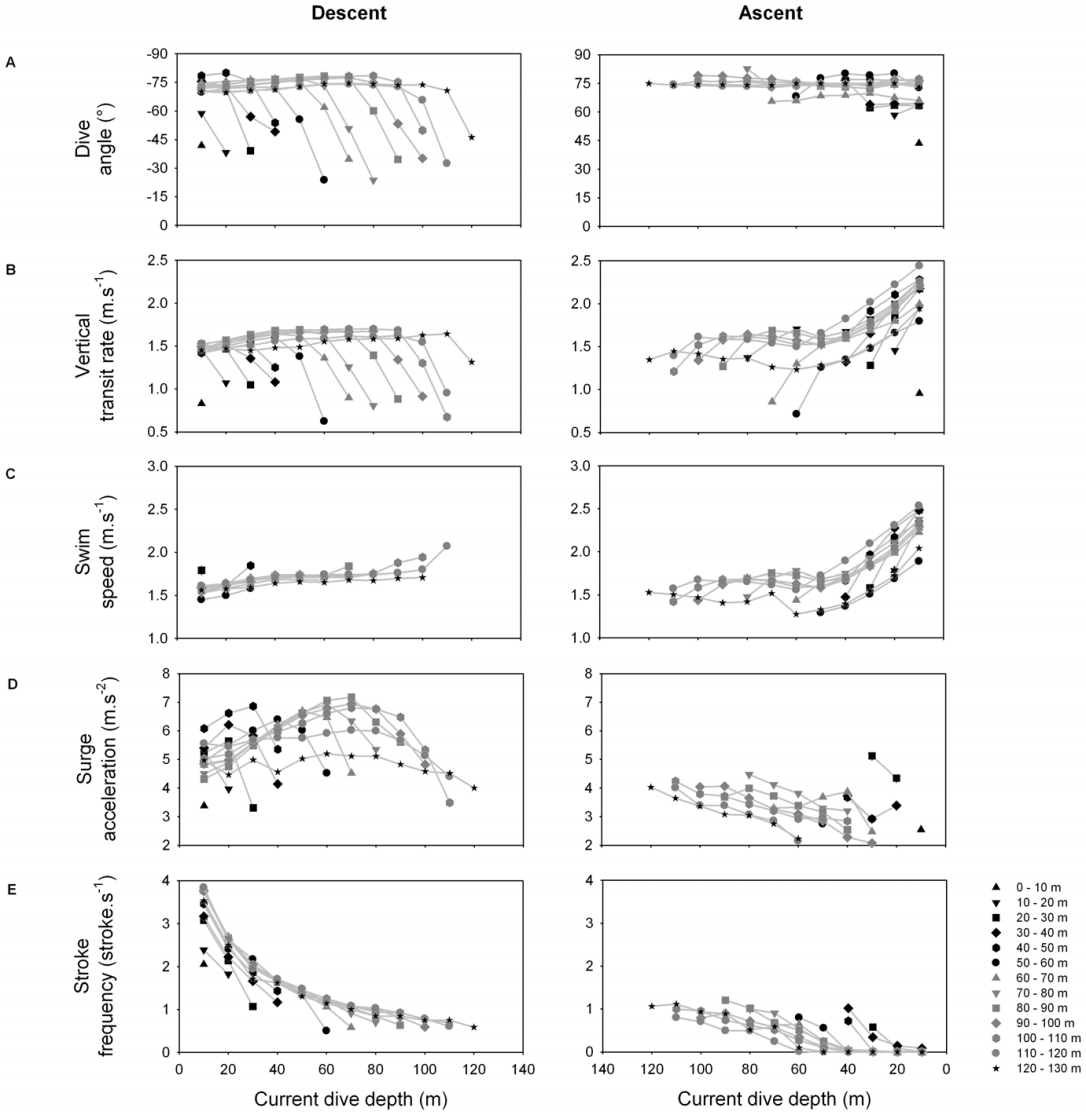
(A) Dive angle, (B) vertical transit rate, (C) swim speed, (D) surge acceleration and (E) foot-stroke frequency, in relation to current dive depth (>5 m), for the descent and the ascent phases and for different maximum dive depth categories. Each value was calculated as the mean of individual means. Because stroking activity is so intense during the first 1–2 s of the descent phase (strong and frequent feet kicks), it was not possible to detect the strokes occurring during this period. Consequently, the first current dive depth category (10 m) ranges from 10–15 m, the second (20 m) from 15–25 m, and so on. A maximum dive depth class stood out (120–130 m); for this class, parameters derive from two dives carried out by the same individual.

**Figure 3:**
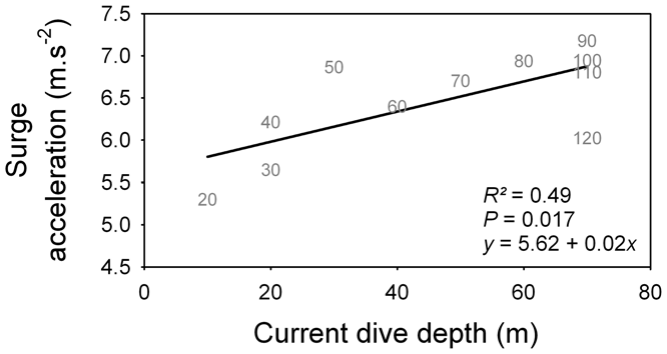
Relationship between peak in surge acceleration for different maximum dive depth categories and current dive depth. For positioning of peaks, see Fig. 2. Each data point is symbolized by a number representing maximum dive depth category. The deepest category (120–130 m) was excluded from the regression (see Fig. 2).

**Figure 4:**
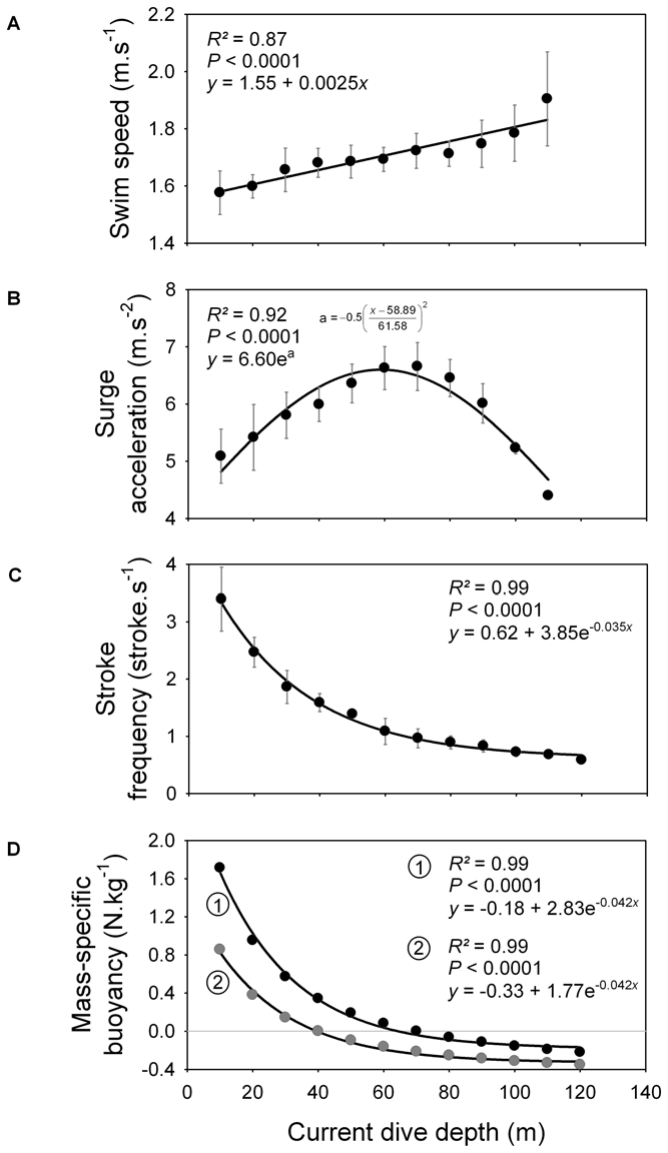
(A) Swim speed, (B) surge acceleration, (C) foot-stroke frequency and (D) mass-specific buoyancy, in relation to current dive depth, for the descent phase. For each current depth class, values of swim speed, surge acceleration and foot stroke frequency were calculated as the mean (± S.D.) of the values calculated for different maximum dive depth categories (Fig. 2). For surge acceleration, the depth class near the end of the descent phase of each maximum dive depth category was excluded because acceleration suddenly decreases as the bird approaches the bottom. The deepest maximum dive depth category (120–130 m) was excluded from the regression (see Fig. 2). For calculation of mass-specific buoyancy, see Appendix S1. Grey circles and black circles correspond to the theoretical buoyancy of birds that load the volume of respiratory air necessary for attaining neutral buoyancy at 40 m and 70 m, respectively. Mass-specific buoyancy at the sea surface is 3.99 N.kg^−1^ and 2.28 N.kg^−1^ for relationship 1 and 2, respectively.

Dive parameters were calculated for both the descent and the ascent phases (Fig. 2). During the descent phase, shags descended almost vertically, as body angle relative to surface was around −75°. Descent angle flattened out as birds approached the bottom of the dive. Surge acceleration increased for all maximum dive depth categories along with current dive depth, reached a peak, and then decreased as birds approached the bottom. The peak occurred at a greater depth as maximum dive depth category increased, and value of surge acceleration at the peak increased with maximum dive depth category (Fig. 2, 3 and 4). Swim speed increased significantly during the descent, from 1.6 to 1.8 m.s^−1^ (Fig. 2 and 4). Stroke frequency decreased exponentially with depth (Fig. 2 and 4).

**Figure 5:**
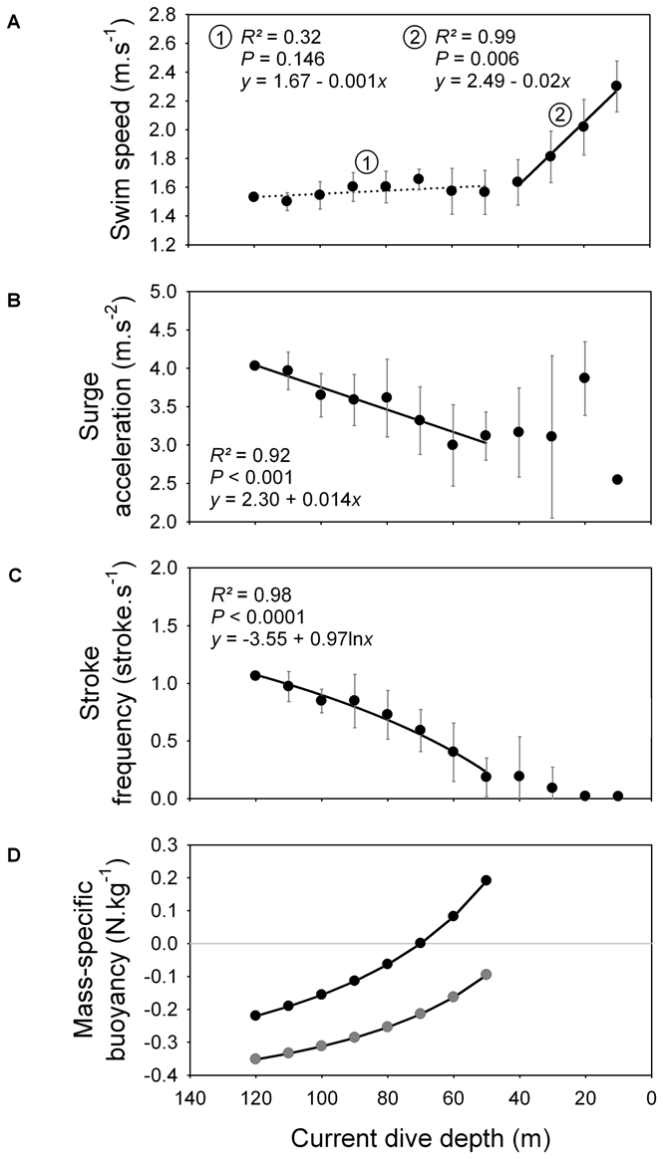
(A) Swim speed, (B) surge acceleration, (C) foot-stroke frequency and (D) mass-specific buoyancy, in relation to current dive depth, for the ascent phase. For each current depth class, values of swim speed, surge acceleration and foot stroke frequency were calculated as the mean (± S.D.) of the values calculated for different maximum dive depth categories (Fig. 2). For calculation of mass-specific buoyancy, see Appendix S1. Grey circles and black circles correspond to the theoretical buoyancy of birds that load the volume of respiratory air necessary for attaining neutral buoyancy at 40 m and 70 m, respectively. Because stroking activity of birds stops at an average depth of 54±11 m for dives >40 m (Fig. 7), the regression for each relationship was only calculated for this first part of the ascent, except for swim speed.

**Figure 6:**
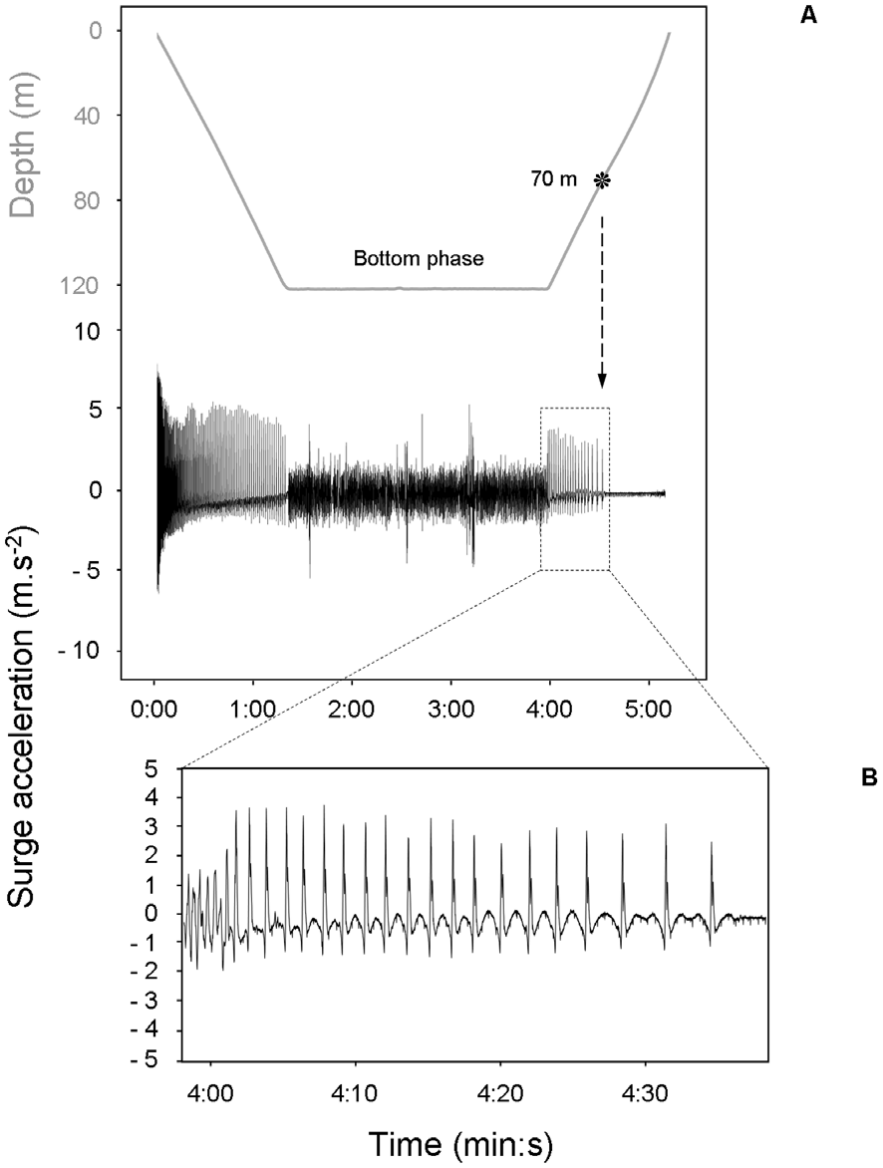
(A) Example for one bird of how paddling activity, represented by surge acceleration, varies in relation to dive phase and (B) close up of the first part of the ascent phase. The vertical excursions during the bottom phase are not similar to the paddling activity that occurs during the descent or the ascent. This noise is the result of a combination of different complex body movements produced by the bird, which include fish hunting and horizontal swimming. Note how stroking activity is intense during the first 1–2 s of descent. In this example, the bird performed 20 foot-strokes to swim from the bottom to a depth of 70 m (each stroke triggered a peak in surge acceleration). The bird reached the surface afterwards through passive rising.

During the ascent phase, shags rose almost vertically (body angle ∼75°). Between 50 and 60 m, birds started gaining momentum rapidly, swim speed increasing from ∼1.6 to almost 2.3 m.s^−1^ close to the sea surface (Fig. 2 and 5). Stroke frequency and surge acceleration decreased with decreasing depth (Fig. 2 and 5). Paddling activity actually disappeared at a depth range of ∼40–70 m (mean: 54±11 m) (Fig. 6). Time spent ascending passively represented 12±8% of total dive duration.

**Figure 7:**
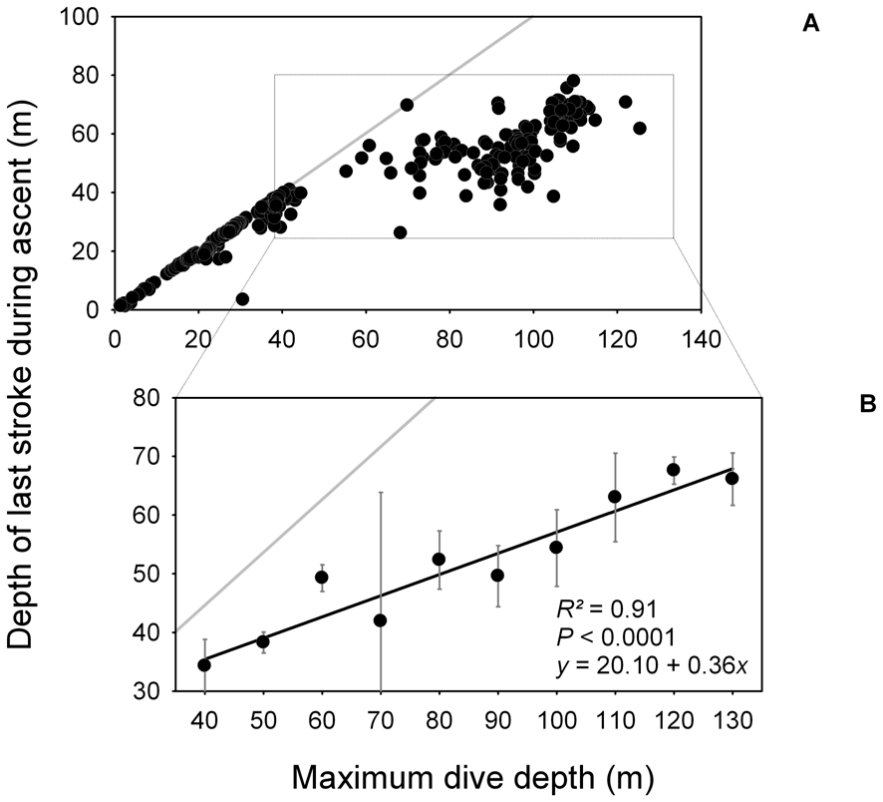
(A) Depth of last foot stroke during ascent in relation to maximum dive depth for all birds and (B) close up for dives strictly >40 m, where depth of last foot-stroke is shallower than maximum dive depth. Because dives are flat-bottomed, maximum dive depth is a good proxy of depth of end of bottom phase (mean difference  = 0.9±1.5 m). For dives <40 m, depth of last foot-stroke during ascent is similar to maximum dive depth, birds gliding from the bottom back to the surface (grey line: *y*  = *x*). For dives >40 m, the depth where the last stroke occurs increases with maximum dive depth (each point is the mean ± S.D. of individual means within each 10 m depth class); the linear regression for this relationship is the black line (grey line: *y*  = *x*).

Maximum dive depth correlated positively with the depth at which the birds stopped swimming during the ascent phase (Fig. 7). The other parameters we tested were dive order and body mass (see Methods). There was no statistical relationship between depth of last foot stroke and dive order, independently of maximum dive depth (no interaction between dive order and maximum dive depth: *F*_1,130_  = 0.34, p  = 0.558). There was also no statistical relationship between depth of last foot stroke and body mass, independently of maximum dive depth (no interaction between body mass and maximum dive depth: *F*_1,130_  = 0.32, p = 0.574).

## Discussion

Our results suggest that Kerguelen shags display behavioural strategies for maximizing the time spent submerged, thus the time spent foraging, relative to O_2_ reserves. We propose that not only do these strategies involve birds constantly adjusting the expenditure rate of their locomotor activity to the natural variation in the force of buoyancy with depth, but they are also characterized by birds probably modifying their own buoyancy intentionally–through respiratory control–as a means for reducing swimming effort.

### Birds adjust their locomotor effort to the natural change of buoyancy with depth

Shags swim almost vertically during both the descent and the ascent phases, consequently minimizing the time of transit between the surface and the feeding grounds and back, thus reducing the O_2_ consumption of transport. Orientation of shags is not strictly vertical, as swimming body angle of birds is generally 15° from this position. The reason for this is not clear. The body angle of actively swimming cormorants is subject to body angle oscillation throughout the paddling cycle [17], [29]. In European shags *Phalacrocorax aristotelis*, the body will eventually deviate from the mean swimming angle by ±15° during the cycle [17]. Because of this body angle oscillation, Kerguelen shags swimming with an mean angle of 90°, instead of 75°, would be alternately swimming with a positive and a negative body angle during the descent and the ascent, respectively. In these conditions, swimming birds would be constantly alternating their heads from an upright to an upside down position throughout transits. Instead, animals might choose to adopt a body angle that is the closest possible to 90°, but which at the same time prevents them from being in an upside down position, a situation that could be disorienting.

Shags enter the water after a pre-dive leap, a small hop above the water surface which, in deep diving foot-propelled divers, enables efficient penetration and immediate submergence of the feet, the organs of locomotion [35]. Just after submergence, shags paddle frequently and strongly (Fig. 2 and 6). This action subsides quickly after 1–2 s, when birds are safely away from the area near the surface, where the force of buoyancy is the strongest.

During the descent, bird swim speed is around 1.7 m.s^−1^ (Fig. 4). This value fits within the standard speed range (1.3–1.8 m.s^−1^) of actively swimming large cormorant species [22], [36], [37], a speed range probably close to optimal swim speed (speed minimizing the cost of transport, [16]) of large cormorants. Because increasing speed also increases drag, swimming faster than the optimal swim speed only leads to exponentially increasing O_2_ consumption. At the same time, stroke frequency decreases according to a pattern that exactly follows the way the force of buoyancy decreases naturally with depth (with a similar exponential decay constant of ∼−0.04, Fig. 4). In parallel, surge acceleration first increases with depth, reaches a peak, and then decreases during the second part of the descent (Fig. 2). Thus, acceleration is relatively low at first (except for the first 1–2 s, see above), because birds are in shallow water and buoyancy is strong. Then acceleration increases; this could be explained by birds stroking more easily with decreasing buoyancy. Eventually, birds decrease acceleration, which could be due to buoyancy being small enough for birds to maintain speed while reducing stroke force and frequency. Near the bottom of the descent, acceleration drops suddenly, probably because birds prepare to approach the sea floor. To summarize, these patterns suggest that while actually increasing their swim speed slightly, birds reduce their expenditure rate, constantly adjusting locomotor activity to decreasing buoyancy. Adjustment of locomotor activity to decreasing buoyancy with depth has also been shown to occur in more shallow-diving species of cormorants [15], [17], [20].

During ascent, shags sometimes cease all locomotor activity and glide to the surface (Fig. 6 and 7). We assumed that birds quit paddling when the upward component of buoyancy was gaining on the force of gravity and that ascent could be done without effort. Beneath the depth where this occurs, swim speed is relatively constant (∼1.6 m.s^−1^, Fig. 5). Concomitantly, surge acceleration and stroke frequency decrease with decreasing depth. Stroke frequency follows a logarithmic decrease function that is opposite to the exponential function guiding the relationship between buoyancy and depth (Fig. 5). Again, birds match their locomotor activity to variation in the force of buoyancy. Here, they use the increasing force of buoyancy to gradually reduce expenditure rate, all the while maintaining optimal swim speed. Cessation of locomotor activity also surely preserves O_2_ saving if birds reach the surface quickly enough in this fashion. This is the case, as birds actually increase values of swim speed substantially compared to the descent phase, just by gliding (Fig. 5). Birds ascending from the bottom when maximum dive depth is <40 m deep rise passively all the way to the surface (Fig. 7). Consequently, they are positively buoyant within this depth range, as has been shown for other species of cormorants diving under 50 m deep [15], [17]. Birds ascending from the bottom phase when maximum dive depth is >40 m deep first reach a certain depth before they cease all foot-stroking activity, and then ascend passively to the surface (Fig. 6 and 7). We propose that for such dives the depth of last foot stroke during the ascent is a proxy for the depth of neutral buoyancy. The actual depth of neutral buoyancy should be slightly deeper, however, because birds must wait for the upward component of buoyancy to become stronger than gravity before giving their last foot-stroke [24].

Thus, Kerguelen shags adjust their buoyancy to maximum dive depth. It has been shown with intermandibular angle sensors (Hall sensors), that the angle of beak opening (a proxy for the volume of inhaled air) in penguins during the final inspiration preceding a dive is related to maximum dive depth [26]. This suggests that penguins can regulate the amount of air they dive with. Kerguelen shags probably also adjust the amount of air they load just before submerging, and this explains variation in buoyancy with dive depth. Certain factors, such as body size, food load, or even plumage wettability may affect buoyancy. However, investigation of these parameters was not conclusive.

### How birds can modify their own buoyancy to reduce foraging effort

Loading more air, therefore more O_2_, upon diving would seem a logical strategy for extending aerobic dive duration [25]. Unfortunately, testing this in the field is actually quite difficult. Shags that dive to a maximum depth of 40 m and 125 m are neutrally buoyant at ∼40 m and 70 m, respectively (Fig. 7). Furthermore, dives reaching a maximum depth of 40 m and 125 m last ∼130 s and 330 s, respectively (Fig. 1). We therefore tested to see if a difference in respiratory air volume (lungs + air sacs) is sufficient to explain variation in depth of neutral buoyancy in Kerguelen shags and the associated differences in dive duration.

Shags are neutrally buoyant at 40 m and 70 m with a respiratory volume of 294 ml and 735 ml (difference: 441 ml), respectively (Appendix S1, Fig. 4 and 5). Considering that 441 ml of air contains 92 ml of O_2_ and that the diving metabolic rate of the Kerguelen shag and other foot-propelled diving seabirds can be estimated as ∼40–90 ml O_2_.min^−1^.kg^−1^ [38], a supplementary 441 ml of respiratory air would only bring an additional 24–53 s of dive duration to a 2.6 kg Kerguelen shag. This is insufficient to explain the 3 min 20 s that separate dives that reach a maximum depth of 40 m and those that reach a maximum of 125 m (Fig. 1). If loading an additional 441 ml of respiratory air upon diving were to explain such an extension in dive duration, diving metabolic rate of Kerguelen shags would have to be  = 10.6 ml O_2_.min^−1^.kg^−1^.

These results should be interpreted cautiously. First, they come from a model (Appendix S1), in which the small modification of a parameter, such as plumage air volume, can change results significantly. In addition, Equation 2 has one weakness, as remaining body volume (*V_T_*) is obtained by subtracting plumage air volume (*V_Fs_*) and respiratory air (lungs + air sacs) volume (*V_Ls_*) from total body volume (*V_B_*). This is due to the difficulty of finding direct estimates of *V_T_* in the literature. Thus, *V_Ls0_* - the parameter which this equation calculates for any maximum dive depth, is somehow already contained within one of the equation's variables. However, this tautology is not insurmountable, as the purpose of the equation is primarily to yield relative values of *V_Ls_* for different maximum dive depths (*V_Ls_* is always relative by definition), but which also fit within a range that is realistic from a morphological point of view. The maximum respiratory (lungs + air sacs) volume of a Kerguelen shag of mass (*m*) equal to 2.6 kg can be calculated as  = 0.126×(10^3^×*m*)^1.12^ = 842 ml (equation for non-galliform birds, [39]), a value which is close to the ones obtained with Equation 2 for Kerguelen shags (735–882 ml) diving in the 130 m range and neutrally buoyant at 70–80 m. This suggests furthermore that shags diving to such depths may be near their maximum respiratory air storage capacity. This illustrates how it is possible for birds to control their buoyancy through respiratory air volume adjustment, and it gives plausible values of air volumes that birds must load in order to do so.

Second, our conclusion that the variations of volume of respiratory O_2_ related to respiration mediated buoyancy controls are insufficient to explain the associated differences in dive duration is based on the paradigm that diving is highly O_2_ consuming for seabirds, particularly when using foot-propulsion (for a review, see [38]). However, respiratory studies rarely duplicate the conditions encountered by divers in the wild. Free-ranging diving seabirds can spend considerable amounts of time in cold water, where they may perform very deep and very long dives. During these periods, many species are thought to be hypometabolic, as they may experience bradycardia or regional hypothermia, or both (for a review, see [40]). Metabolic depression should enable long periods of submergence with a purely aerobic metabolism. In this context, an increase in the respiratory O_2_ load upon diving would make it possible for the bird to increase dive duration substantially. However, if the O_2_ stored in the blood and the skeletal muscles of cormorants represents 60–80% of the total body O_2_ volume, as in penguins [2], [41], and if Kerguelen shags also rely on anaerobic metabolism to some extent for deep diving [42], the relative contribution of respiratory O_2_ to long dive durations should, in any case, be limited. Therefore, although it may indeed help to extend dive duration to a certain extent, increasing the volume of respiratory O_2_ upon diving is probably insufficient, in itself, to explain some of the dive durations recorded here in the Kerguelen shag (almost 6 min).

Consequently, unless previous estimates of blood and skeletal O_2_ stores and diving metabolic rates in avian divers are highly over-estimated, a more important advantage of respiratory air volume adjustment in cormorants, as in perhaps other families of diving seabirds, would be to reduce the costs of buoyancy control. For penguins, it has also been suggested that they optimise the costs and benefits of buoyancy through respiration mediated buoyancy control [11], [26]. In many situations, the benefits of increasing the respiratory O_2_ load to increase dive duration slightly could be quickly outweighed by the associated locomotor costs of fighting against higher buoyancy during the descent and the bottom phase of the dive. For example, smaller air loads should be an advantage during the descent and the bottom phase of shallow dives. In benthic diving species in particular, reducing buoyancy at the bottom (bottom phase: 40–90% of dive duration in the Kerguelen shag, Table 1) of shallow dives should help birds hunt prey with minimum expense, because they are not be struggling to stay at depth or maintain control of roll, pitch and yaw (‘minimal buoyancy strategy’, [26]). Because maximum dive depths of successive dives are often autocorrelated in benthic divers [43], animals should be capable of finely adjusting the volume of respiratory air they dive with, based on the efficiency of their previous dive [25]. On the same principle, increasing respiratory air loads, therefore buoyancy, at the bottom of deep dives would be beneficial because it would prevent birds from sinking and subsequently wasting the O_2_ necessary to fight for control. Furthermore, the deeper they dive, the greater the depth from which Kerguelen shags ascend to the surface without paddling (Fig. 6 and 7). Thus, birds seem to use a ‘maximal buoyancy strategy’ for deep dives, a behaviour which would save oxygen not only during the bottom phase, but also during the ascent phase. Eventually, although increasing respiratory O_2_ volume when diving deeper seems to have a limited effect on overall aerobic dive duration (see above), its importance should not be entirely discarded, particularly as it may pay off the inevitable increase in the cost of transport of the descent phase associated to greater respiratory air loads in deep dives.

### Conclusion

To conclude: 1) diving Kerguelen shags adjust their locomotor activity to the natural variation of buoyancy with depth, thus saving energy, 2) they stop foot stroking at one point during ascent, passively gaining the surface from there on, and the depth at which they do so is positively related to maximum dive depth, 3) depth of last foot stroke can be considered as a proxy for the depth of neutral buoyancy, and this suggests that the air volumes loaded by birds are larger when they target deeper depths, 4) the most parsimonious explanation for this is that birds have a respiration mediated buoyancy control, and 5) simple calculations suggest that, unless known figures for blood and skeletal muscles oxygen stores and avian diving metabolic rates are highly overestimated, the benefits of respiration mediated buoyancy control are related less to increasing body O_2_ stores when diving deeper, therefore longer, than to reducing the costs of locomotor buoyancy control at all depths. Here, we provide empirical data and a conceptual framework to explore the question of the relationship between respiratory volume, dive duration and dive depth. A depth-dependant diving metabolic rate modelling approach (taking into account other complex variables, such as drag [44]), will be necessary to untangle the different costs and benefits of respiratory and underwater locomotor strategies of avian divers.

## Supporting Information

Appendix S1 Equations for calculating the force of buoyancy as a function of depth and the respiratory volume (lungs + air sacs) as a function of depth of neutral buoyancy in diving birds.
(0.03 MB DOC)
