# Supplementary material for: Buoyancy under Control: Underwater Locomotor Performance in a Deep Diving Seabird Suggests Respiratory Strategies for Reducing Foraging Effort
Source: PLoS One. 2010 Mar 23;5(3):e9839. doi: 10.1371/journal.pone.0009839 (PMC2843744; doi:10.1371/journal.pone.0009839)
Supplement: Appendix S1 — Equations for calculating the force of buoyancy as a function of depth and the respiratory volume (lungs + air sacs) as a function of depth of neutral buoyancy in diving birds. (0.03 MB DOC) [file pone.0009839.s001.doc]

**Appendix S1 - Equations for calculating the force of buoyancy as a function of depth and the respiratory volume (lungs + air sacs) as a function of depth of neutral buoyancy in diving birds**

**Equation 1: the force of buoyancy (‘net buoyancy’) as a function of depth in a diving bird (from [1])**

*F_UP_*: buoyancy (N) at any *d*

*F_UPms_*: mass specific buoyancy (N.kg^-1^) = *F_UP_*/*m*

*d*: current dive depth (m)

*m*: body mass of bird (kg) = 2.6 kg for a Kerguelen shag (mean value)

*ρ*: density of water (kg.m^-3^) = 1,027 kg.m^-3^ for seawater at Kerguelen in summer (temperature: 4 ºC, salinity: 34 g.kg^-1^, [2])

*g*: gravitational acceleration (m.s^-2^) at sea level = 9.807 m.s^-2^

*p_s_*: standard atmospheric pressure (Pa) at sea level = 101,325 Pa

*V_B_*: total body volume (m^3^) of bird at sea level = *m*/(0.81×10^3^) = 0.003210 m^3^ for a Kerguelen shag (inferred from values for the great cormorant *Phalacrocorax carbo*: [3,4])

*V_Ls_*: volume (m^3^) of respiratory system (lungs + air sacs) of bird at sea level (arbitrarily chosen as *V_Ls_* = 1.61×10^-4^×*m*^0.91^, [5]) = 0.000384 m^3^ for a Kerguelen shag

*V_Fs_*: volume (m^3^) of air contained in the plumage of bird at sea level = *m*×(0.17×10^-3^) for a great cormorant *Phalacrocorax carbo* [6] = 0.000442 m^3^ for a Kerguelen shag

*V_T_*: volume (m^3^) of the remaining body tissue of bird = *V_B_* - *V_Ls_* - *V_Fs_* = 0.002384 m^3^ for a Kerguelen shag (this *V_T_* implies a density of remaining body tissue of 1090 kg.m^-3^, which is greater than the mean value of 1025 kg.m^-3^ estimated after dissection of one great cormorant *Phalacrocorax carbo* and one Cape cormorant *Phalacrocorax capensis* by [1])

$$F_{UP}=\rho g\left(\frac{p_{s}\left(V_{Ls}+V_{Fs}\right)}{p_{s}+\rho gd}+V_{T}\right)-gm$$

**Equation 2: the respiratory volume (lungs + air sacs) as a function of depth of neutral buoyancy in a diving bird (derived from Equation 1 by constraining F_UP_ = 0)**

*V_Ls0_*: *V_Ls_* necessary for *F_UP_* = 0 at *d*_0_

*d*_0_: depth where *F_UP_* = 0

$$V_{Ls0}=gd_{0}\left(\frac{m-\rho V_{T}}{p_{s}}\right)+\left(\frac{m}{\rho}\right)-V_{T}-V_{Fs}$$

In the Kerguelen shag, *V_Ls0_* = 0.000294 m^3^ and 0.000735 m^3^ for *d*_0_ = 40 m and 70 m, respectively

*F_UP_* can be calculated as a function of *d* for any *d*_0_, by replacing *V_Ls_* in Equation 1 by *V_Ls0_* found in Equation 2. If Kerguelen shags reach their maximum *V_Ls_* capacity for *d*_0_ = 70 - 80 m (see text), *F_UP_* is a function of *d* only for *d*_0_ ≤ 70 - 80 m

**References**

1. Wilson RP, Hustler K, Ryan PG, Burger AE, Nöldeke EC (1992) Diving birds in cold water: do Archimedes and Boyle determine energetic costs? Am Nat 140: 179–200.

2. Fofonoff P, Millard RC (1983) Algorithms for computation of fundamental properties of seawater. Unesco Tech Pap Mar Sci 44: 1–53.

3. Lovvorn JR, Jones DR (1991) Body mass, volume, and buoyancy of some aquatic birds and their relation to locomotor strategies. Can J Zool 96: 2888–2892.

4. Ribak G, Weihs D, Arad Z (2004) How do cormorants counter buoyancy during submerged swimming? J Exp Biol 207: 2101–2114.

5. Lasiewski RC, WA Calder (1971) A preliminary allometric analysis of respiratory variables in resting birds. Resp Physiol 11: 152–166.

6. Grémillet D, Chauvin C, Wilson RP, Le Maho Y, Wanless S (2005) Unusual feather structure allows partial plumage wettability in diving great cormorants *Phalacrocorax carbo*. J Avian Biol 36: 57–63.
